# Mapping the lignin distribution in pretreated sugarcane bagasse by confocal and fluorescence lifetime imaging microscopy

**DOI:** 10.1186/1754-6834-6-43

**Published:** 2013-04-01

**Authors:** Vitor Carlos Coletta, Camila Alves Rezende, Fernando Rodrigues da Conceição, Igor Polikarpov, Francisco Eduardo Gontijo Guimarães

**Affiliations:** 1 Instituto de Física de São Carlos, Universidade de São Paulo, Caixa Postal 369, São Carlos, SP, CEP 13560-970, Brazil

**Keywords:** Ethanol, Sugarcane, Bagasse, Pretreatments, Cellulose, Lignin, Fluorescence, Microscopy

## Abstract

**Background:**

Delignification pretreatments of biomass and methods to assess their efficacy are crucial for biomass-to-biofuels research and technology. Here, we applied confocal and fluorescence lifetime imaging microscopy (FLIM) using one- and two-photon excitation to map the lignin distribution within bagasse fibers pretreated with acid and alkali. The evaluated spectra and decay times are correlated with previously calculated lignin fractions. We have also investigated the influence of the pretreatment on the lignin distribution in the cell wall by analyzing the changes in the fluorescence characteristics using two-photon excitation. Eucalyptus fibers were also analyzed for comparison.

**Results:**

Fluorescence spectra and variations of the decay time correlate well with the delignification yield and the lignin distribution. The decay dependences are considered two-exponential, one with a rapid (τ_1_) and the other with a slow (τ_2_) decay time. The fastest decay is associated to concentrated lignin in the bagasse and has a low sensitivity to the treatment. The fluorescence decay time became longer with the increase of the alkali concentration used in the treatment, which corresponds to lignin emission in a less concentrated environment. In addition, the two-photon fluorescence spectrum is very sensitive to lignin content and accumulation in the cell wall, broadening with the acid pretreatment and narrowing with the alkali one. Heterogeneity of the pretreated cell wall was observed.

**Conclusions:**

Our results reveal lignin domains with different concentration levels. The acid pretreatment caused a disorder in the arrangement of lignin and its accumulation in the external border of the cell wall. The alkali pretreatment efficiently removed lignin from the middle of the bagasse fibers, but was less effective in its removal from their surfaces. Our results evidenced a strong correlation between the decay times of the lignin fluorescence and its distribution within the cell wall. A new variety of lignin fluorescence states were accessed by two-photon excitation, which allowed an even broader, but complementary, optical characterization of lignocellulosic materials. These results suggest that the lignin arrangement in untreated bagasse fiber is based on a well-organized nanoenvironment that favors a very low level of interaction between the molecules.

## Background

First generation ethanol is currently produced in large-scale in Brazil by the fermentation of sugarcane juice. The solid residue obtained after the juice extraction, known as bagasse, is an important renewable energy source, containing 60% to 80% of carbohydrates on a dry-matter basis [1,2]. Bagasse is, therefore, a promising feedstock with a potential to be used for cellulosic ethanol production, promoting greater ethanol yields per hectare in a sustainable and environmental-friendly manner.

Plant cell wall is a complex array, consisting mainly of a mix of crystalline and amorphous cellulose, surrounded by hemicellulose and by lignin [3,4]. The production of second generation biofuels from bagasse and other lignocellulosic residual materials relies on pretreatments to separate the cell wall components and on the conversion of cellulose and part of the hemicellulose into fermentable sugars, which can be performed by enzymatic hydrolysis [4,5].

The major drawback for this biofuel production route is the cell wall recalcitrance, strongly related to its lignin content and distribution. Lignin is a phenolic, branched and hydrophobic polymer that forms a reinforced network on the cell wall and interferes with the enzyme action by decreasing cellulose accessibility or by unproductive adsorption. It is highly resistant to physical, chemical and biological degradation, and it is, thus, not degraded by the enzymes that hydrolyze cellulose [4,6,7].

The viability of cellulosic ethanol production depends on the development of effective pretreatment technologies to promote sample delignification with minimal carbohydrate degradation and no harm for hydrolysis or fermentation [8,9]. The role of pretreatment is to improve hydrolysis yields by separating the biomass contents, removing lignin, and favoring the enzyme access to cellulose [5,10]. Acid treatments have been considered effective to hydrolyze hemicellulose [11-14], whereas alkali treatments are useful to remove lignin [8,15,16].

In our previous work, we applied a two-step pretreatment, using acid and alkali, and investigated the modifications of the morphology and chemical composition of sugarcane bagasse samples under various alkali concentrations [1]. High proficiency liquid chromatography (HPLC) and UV–vis spectroscopy were used to obtain detailed sample composition, considering cellulose, hemicellulose, lignin and ash amounts. Nuclear magnetic resonance (NMR) spectra were also recorded and the reduction on the intensity of the lines assigned to hemicellulose and lignin chemical groups confirmed the effectiveness of the treatment [1]. However, NMR produced mostly qualitative results.

Furthermore, no information concerning local distribution of lignin through the cell-wall matrix has been obtained. This is a very relevant issue, however, because the biomass recalcitrance is determined not only by the lignin content, but also by its dispersion within the matrix. Samples with similar lignin amounts may present distinct hydrolysis behaviors, as a consequence of different lignin distributions.

Confocal laser scanning microscopy (CLSM) and fluorescence lifetime imaging microscopy (FLIM) are able to provide important information about the concentration and the spatial distribution of a fluorophore within a sample, since this molecule has a characteristic fluorescence decay time depending on the microenvironments [17]. Regarding pretreatment effects in biomass, CLSM was previously used to investigate lignin degradation [18,19] and redistribution [20], xylan redistribution [21], cell wall swelling [22] and cellulose exposure [23]. Furthermore, an application of FLIM to lignocellulosic materials was previously performed by Hafrén and Oosterveld-Hut, who showed the influence of photobleaching on the fluorescence decay time distributions of thermomechanical pulp paper [24]. The use of FLIM allows one to map the lignin distribution along the cell wall of a single lignocellulosic fiber submitted to an alkali treatment, since the optical processes associated to the remaining lignin are strongly dependent on the lignin concentration rather than on its chemical modifications.

In this paper, we used confocal and FLIM images using one-photon (1P) and two-photon (2P) excitation to quantify lignin fraction and distribution along single fibers of sugar cane bagasse after the pretreatment with H_2_SO_4_ and NaOH. A lignin film and delignified eucalyptus fibers were used as examples of highly lignified and delignified samples, respectively, for comparison with bagasse single fibers treated with acid and alkali. A direct linear correlation between fluorescence decay times and lignin fraction in the bagasse cell wall was obtained and can be used as a reproducible method to follow and determine lignin content after bagasse pre-treatments.

## Results and discussion

**Figure 1:**
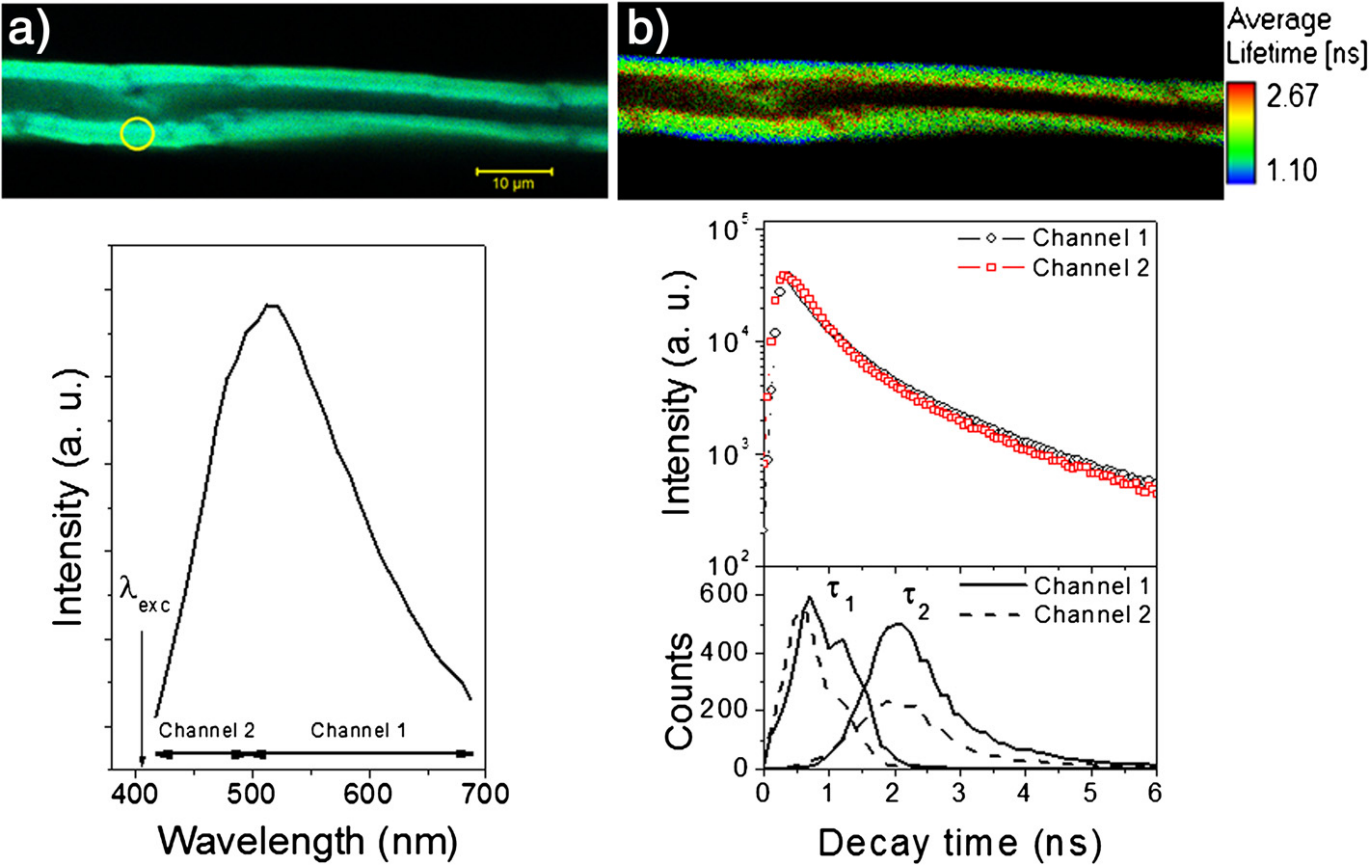
**Confocal and FLIM images for a single bagasse fiber: spectral and fluorescence decay features. a**) Spectral confocal image of a single bagasse fiber treated with NaOH 0.5% excited at λ_exc_ = 405 nm (continuous wave). The spectrum below corresponds to the emission evaluated at the yellow spot of the cell wall. The spectral regions for channels 1 and 2 used in the decay time measurements are displayed in the figure. **b**) The corresponding FLIM image and the associated decay features detected from channels 1 and 2. The figure below shows the decay time distributions for τ_1_ and τ_2_ evaluated from the FLIM image for channel 1 (solid lines) and channel 2 (dashed lines).

Figures 1(a) and 1(b) exemplify confocal images in the spectral and FLIM modes, respectively, for a single bagasse fiber treated with NaOH 0.5% and their corresponding fluorescence spectra and time decay along the fiber. The broad emission spectrum evaluated at a position (yellow circle) on the cell wall is basically due to lignin excited by continuous wave (CW) 1P illumination at 405 nm. This wavelength corresponds to optical transitions involving the low energy tail of the absorption band states that do persist over a wide spectral range (much higher than 400 nm) [25,26], due to the great inhomogeneity of lignin in the complex sugarcane structure.

The detection of the fluorescence decay in two spectral ranges is very convenient for studying very heterogeneous materials due to their characteristic broad emission. Properties like decay time may depend on the detected wavelength. If not, we can detect longer wavelength (channel 1) to avoid emission reabsorption and scattering effects along the optical path inside of the cell wall (see discussion in the following). Taking into account this broad emission band, the dynamics of the excited state was probed by taking the fluorescence decay for two distinct spectral ranges: above the emission maximum around 490 nm (channel 1) and below this wavelength (channel 2). The corresponding FLIM image of Figure 1a and the associated emission decay features integrated for all pixels of channels 1 and 2 are presented in Figure 1b. This figure shows that there is no significant difference in the decay characteristics of channels 1 and 2 when the excitation of a pulsed laser at 405 nm is used. The fluorescence time decay for lignin can be readily fitted by superposing two exponentials, one with a rapid (τ_1_) and the other with a slow (τ_2_) decay time. The frequencies of τ_1_ and τ_2_ are given by their respective distributions (Figure 1b, bottom) that were estimated from the decay behavior of each pixel for channels 1 (solid lines) and 2 (dashed lines) in the FLIM image. It is worth noting that these distributions present almost the same features for channels 1 and 2, but for the slow and the rapid components are well separated in time.

**Figure 2:**
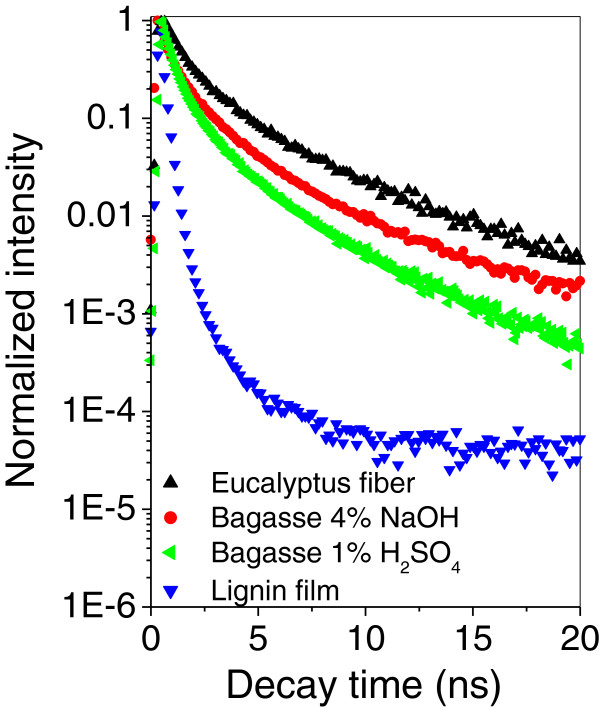
**Fluorescence decay behavior for lignin in different systems.** Comparison among the fluorescence decay dependences evaluated from single fiber FLIM images of bagasse treated with H_2_SO_4_ 1%, bagasse treated with NaOH 4%, eucalyptus fiber and lignin film.

Figure 2 shows how the fluorescence decay changes for different samples excited by a pulsed laser light at 405 nm in the nanosecond range. For better viewing in the figure, decays are displayed only for a few selected samples. It includes the total decay profiles resulting from single fiber FLIM images of bagasse treated with H_2_SO_4_ 1% and NaOH 4%, as well as from delignified eucalyptus pulp and from a lignin film that were used as reference. It is clear from this figure that the fluorescence decay behavior depends strongly on the sample nature and on the pretreatment applied to the biomass. Since the pretreatments are used for removing hemicellulose and lignin, there is also a correlation between the fluorescence decay and the lignin content on the samples. The decay is faster for a dense lignin film in Figure 2 and becomes slower on the other samples, the lower their lignin contents are.

**Figure 3:**
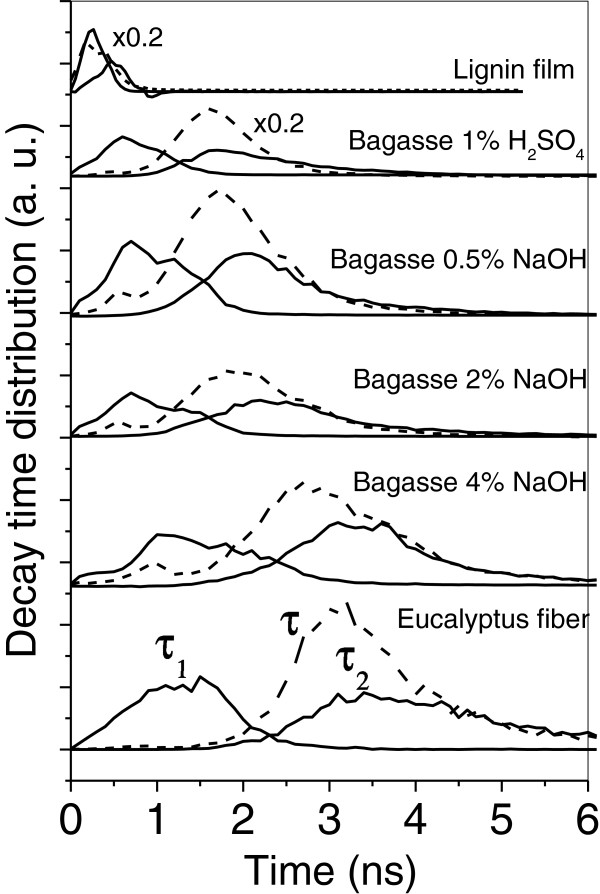
**Effect of NaOH treatment on the decay time distribution.** Rapid (τ_1_) and slow (τ_2_) decay time distributions evaluated from FLIM image and the corresponding average decay time (τ) for bagasse treated with H_2_SO_4_ 1%, NaOH 0.5%, 2% and 4%. The same distributions are also presented for the eucalyptus fiber and the lignin film for comparison. The factor 0.2 was used just to set the counts to a convenient value just to get a better comparison among distributions.

The variation of the decay time can be used to determine the lignin content and distribution along the cell wall and also to correlate the yield of delignification with a specific pretreatment process. Here, the decay time distributions for τ_1_ and τ_2_ and the mean decay time τ can be used to exemplify this methodology. Figure 3 shows the effect of the H_2_SO_4_ and NaOH pretreatments on the decay time distributions of the bagasse samples. The distributions for the reference lignin film and the highly delignified eucalyptus fiber (lignin content under 2%) are also presented for comparison. It is evident that there is a significant shift of the distributions for τ_1_, τ_2_ (solid lines) and τ (dashed lines) to higher decay times with the increase in the NaOH concentration in the alkali pretreatment. This shift is strongly correlated to the delignification process since NaOH treatments are known for removing lignin from biomass [8,15,16] and result in a considerable decrease of the lignin concentration in the studied samples [1]. The lower lignin contents in the fibers are thus associated to slower decays. The position of the distributions at the low decay time range for the sample treated with H_2_SO_4_ suggests that this pretreatment alone is not so efficient for lignin removal, as it has been observed previously in our studies [1]. Furthermore, the position of the distributions for the reference samples is also consistent with our findings concerning the lignin content in the bagasse samples studied here: the dense lignin film presents decay times in the subnanosecond range, while the decay times for the eucalyptus pulp fiber, which is almost lignin free, spread over the range from 1 to 6 ns. It is interesting to note that, besides the shift to higher decay times, the distributions for τ_1_, τ_2_ and τ also become considerably broader with the increase of the NaOH concentration used in the pretreatments. Since FLIM evaluates the decay time all over the fiber, the broadening of the FLIM distributions is an indication of a disordering event taking place due to the lignin rearrangement in the biomass after H_2_SO_4_ pretreatment or after the subsequent treatment with increasing NaOH concentrations. Changes on the chemical environment or structural modifications of lignin would generate this disorder, since each pixel in the FLIM methodology is able to probe the submicroscopic structure of the cell wall through the lignin emission.

**Figure 4:**
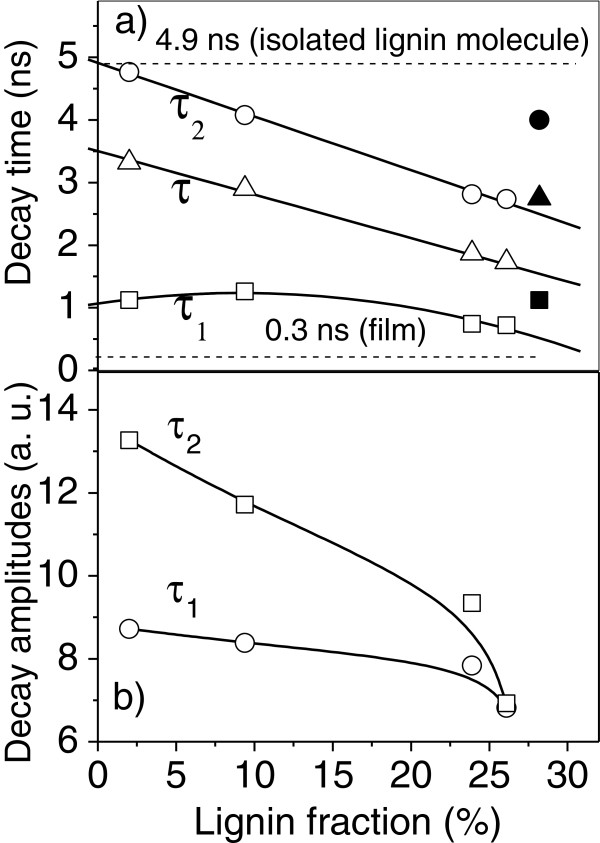
**Decay time and distribution area as a function of lignin content. a**) Dependence of rapid (τ_1_), slow (τ_2_) and average (τ) decay times (open symbols) on the lignin fraction for bagasse fibers with lignin contents between 9 and 28% for an eucalyptus fiber with lignin concentration of 2%. Linear regression curves were fitted to τ_2_ and τ dependences on the lignin content, whereas the saturation behavior for τ_1_ cannot be properly adjusted by a function that could express some theoretically based model. The corresponding decay time values (filled square, circle and triangle) for untreated bagasse fibers are also shown separately. A decay time of 4.9 ns was estimated for a non-interacting lignin molecule by extrapolating the τ_2_ linear dependence for zero lignin fraction. For lignin in a dense film, the decay was estimated to be around 0.3 ns. **b**) Area below the τ_1_ and τ_2_ distributions as a function of the lignin fraction.

The total decay features integrated for all pixels of the FLIM image (see Figure 2) produce good statistics for reliable mean values of τ_1_ and τ_2_, which would overcome the strong spreading of these quantities due to the disorder introduced by the treatments. Figure 4a shows these decay times (open squares for τ_1_ and circles for τ_2_), now as a function of the lignin concentrations obtained from HPLC data (Table 1) for bagasse pretreated with H_2_SO_4_ and with NaOH. These lignin fractions were calculated without considering the ashes as part of bagasse, since they represent mainly impurities accumulated before the pretreatment [1].

**Table 1:** Chemical composition of the untreated bagasse sample and samples that underwent acid and alkali pretreatments

**Bagasse samples**	**Bagasse composition (%)**			
	**Cellulose**	**Hemicellulose**	**Lignin**	**Total**
Untreated	44.5 ± 1.1	31.0 ± 0.8	28.2 ± 0.1	103.7 ± 3.5
H_2_SO_4_ 1%	58.3 ± 0.2	8.9 ± 0.8	33.6 ± 0.7	100.8 ± 1.7
NaOH 0.25%	68.3 ± 0.5	5.4 ± 0.1	26.0 ± 0.3	99.7 ± 0.9
NaOH 0.5%	71.3 ± 1.4	3.5 ± 0.2	24.0 ± 6.9	98.7 ± 0.4
NaOH 1%	83.2 ± 0.6	3.2 ± 0.1	11.2 ± 0.9	97.6 ± 1.2
NaOH 2%	85.9 ± 0.3	3.4 ± 0.1	9.6 ± 0.5	98.8 ± 1.1
NaOH 3%	87.2 ± 0.1	3.2 ± 0.1	9.7 ± 0.5	100.1 ± 0.4
NaOH 4%	84.9 ± 3.9	3.3 ± 0.1	9.4 ± 0.4	97.6 ± 4.7

Contents are expressed on a bagasse dry weight basis as an average (± standard deviation) of duplicate determinations. Percentages were calculated by discounting the ash amounts on each sample.

The value of the slow component of the decay (τ_2_), as well as the mean decay time τ decrease linearly as the lignin content of the bagasse increases, while the rapid component (τ_1_) shows a small variation that saturates at about 1 ns in the range of low lignin contents. This behavior for τ_1_ is adjusted by a polynomial function, although it does not express any theoretically based model. The extrapolation of τ_2_ linear dependence to its value for a totally delignified bagasse gives an upper limit for the lignin fluorescence life time close to 5 ns (Figure 4a), which is assigned here to the mean decay time of a non-interacting (isolated) molecule. The value of 0.3 ns sets a lower limit for the decay time in a closely packed lignin material, such as the lignin constituent of the film.

Lignin concentrations lower than 9% could not be obtained when alkaline concentrations higher than 1% were employed to sugar cane pith, since we are in the upper limit of lignin removal from these samples, as demonstrated in Reference [1]. So, only few points (lignin fractions for bagasse pith of 26%, 23% and 9%) are included in Figure 4. Although the decay times still changes for alkaline concentrations higher than 1% for bagasse single fibers, as shown is Figure 3, the correlation with lignin content could not be done. Reference samples were then used to expand the lignin range, as it was the case for the bleached eucalyptus sample containing nominally 2% of lignin. In spite of the substrate changes in the lower limit of lignin amounts, the correlation was still observed.

The amplitude of the slow and the rapid exponential decays also provides the weight of each decay component to the total fluorescence decay of a FLIM image. Figure 4b gives the dependence of the amplitude of each exponential associated to τ_1_ and τ_2_ on the lignin fraction. Both exponential components have almost the same contribution to the total fluorescence decay for lignin fractions close to 30%, the naturally occurring value found in the sugarcane species studied here. For a further decrease in the lignin concentration, the weight of the slow component (τ_2_) steady increases while it saturates for the rapid component (τ_1_). This behavior is consistent with the trend that longer decay times dominate the decay process in the range of lower lignin concentration.

Figure 4a also depicts separately the values for τ_1_ = 1.3 ns (filled square) and for τ_2_ = 4 ns (filled circle) for untreated bagasse fibers. These decay times are very close to the values obtained for highly delignified fibers, which seems to be contradictory, since lignin fraction is expected to be around 30% for the raw bagasse. However, our data provides fundamental evidence for understanding the lignin structure in the sugarcane bagasse. Although highly concentrated lignin molecules are distributed throughout the original cell wall of sugarcane, they are arranged in a much less self-interacting (less concentrated) way in the pristine biomass, which is in agreement with the tridimensional network formed by crosslinked lignin on the cell wall structure. This is consistent with the trend that τ_1_ and τ_2_ present much higher values for sugarcane bagasse than those measured in a dense lignin film. In addition, the two distinguishable distributions for rapid (τ_1_) and slow (τ_2_) decays suggest that there are different domains in the cell wall with very different lignin contents. The low sensitivity of the rapid component τ_1_ to delignification below the total lignin level of 23% (Figure 4a) reinforces the assumption that there are cell-wall domains where lignin is more concentrated, where NaOH does not penetrate. Conversely, in other regions, where lignin is highly sensitive to the alkali treatment, less concentrated lignin is expected (slow τ_2_).

The displacement of the decay times (Figure 4a) and the variations of their amplitudes (Figure 4b) can be associated to changes in the excited state (exciton) dynamics. This is assigned mostly to the rearrangement and the removal of lignin rather than to the chemical modification of this molecule, caused by the NaOH pretreatment. This statement is consistent with the fact that a considerable fraction of the lignin has been removed by the alkali pretreatment under the conditions applied in this study. Moreover, closely packed molecular arrangements, as those found in the lignin film, favor long range dipole-dipole interactions that, consequently, introduces additional energy transfer channels that compete with its internal relaxation and emission. These concurrent processes are also responsible for exciton migration among lignin molecules, which also increases the probability of quenching at non-radiative sites. All these competing mechanisms raise substantially the exciton radiative decay rate. On the other hand, NaOH pretreatments remove lignin, reducing competing non-radiative channels and migration, which may extend the radiative decay rate.

So far, we have used one-photon (1P) to excite lignin fluorescence in the fibers. Moreover, the absorption of lignin macromolecules is characterized by a wide tail of states, ranging from the near ultraviolet to the visible, due to their complexity, degree of polymerization and diversity. As a consequence, an intense and broad luminescence spectrum can still be generated by using photon excitation along the absorption band tail. However, transitions between states of the same parity, which are forbidden for one-photon excitation, are allowed for the two-photon (2P) ones [27]. This suggests that a new variety of states can be accessed by two-photons in order to get an even broader, but complementary, characterization of lignocellulosic materials.

**Figure 5:**
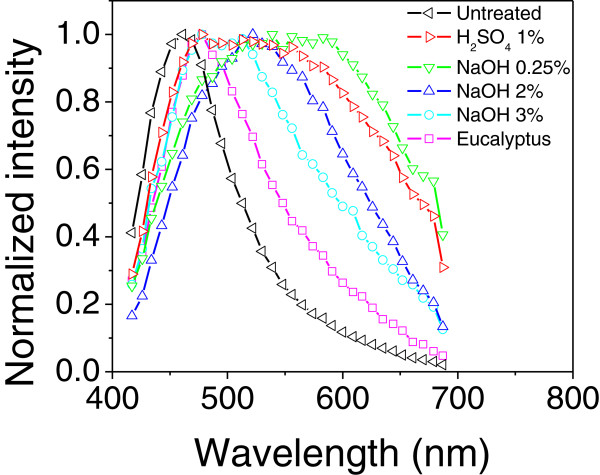
**Fluorescence spectra for two-photon excitation.** Dependence of the emission spectra on different treatment processes. Samples were excited by two photons (2P) at 770 nm. The spectra were normalized at the spectral emission maximum for each sample just for comparison. Some concentrations were omitted in order to better display the behavior for which the displacement of the emission bands were consistent with the alkaline deslignification process.

Figure 5 shows the fluorescence spectra for bagasse with different pretreatments and for the delignified eucalyptus pulp as reference fiber, which were excited with 2P excitation at 770 nm. The 2P emissions of pretreated bagasse are characterized by a much broader line shape than the one observed for 1P excitation (see Figure 1). As the spectra exceeded the detection range of the spectrometer used for this experiment, we normalized them at the intensity maximum in order to highlight changes in the emission line-width and displacement. It is very interesting to see that the untreated bagasse has an unexpected narrow 2P emission band centered at the blue spectral range. However, the emission broadens considerably in the range of high wavelengths comprising the visible and the near infrared region. This is a clear indication that the natural lignin arrangement in the untreated bagasse has been strongly changed by the acid treatment, in such a way that new 2P excited states are formed in the very-low-energy tail of lignin density of states.

The increased number of different local environments around each molecule would explain such a strong spectral modification [28]. Great disorder may occur during acid pretreatment because solubilized lignin molecules react with monomers and oligomers to form larger molecules [29], thus affecting the molecular structure of this polymer and its stable or metastable conformations at nanoscale [7]. For the samples treated with NaOH 0.25%, the broadening still persists, but an increase in alkali concentration causes the narrowing of the 2P spectra in the visible range. This is a convincing evidence that polymer molecules are becoming less concentrated and being removed from the cell wall. Thus, the delignification process eliminates 2P excited aggregated states and decreases the probability of energy migration between them, which results in a displacement of the spectrum to shorter wavelengths. Consistently, the reference spectrum of the highly delignified eucalyptus fiber also presented further narrowing. The narrow spectrum measured for the raw bagasse fiber indicates that the lignin arrangement is based on a well-organized nanoenvironment that favors a very low level of interaction between the molecules.

**Figure 6:**
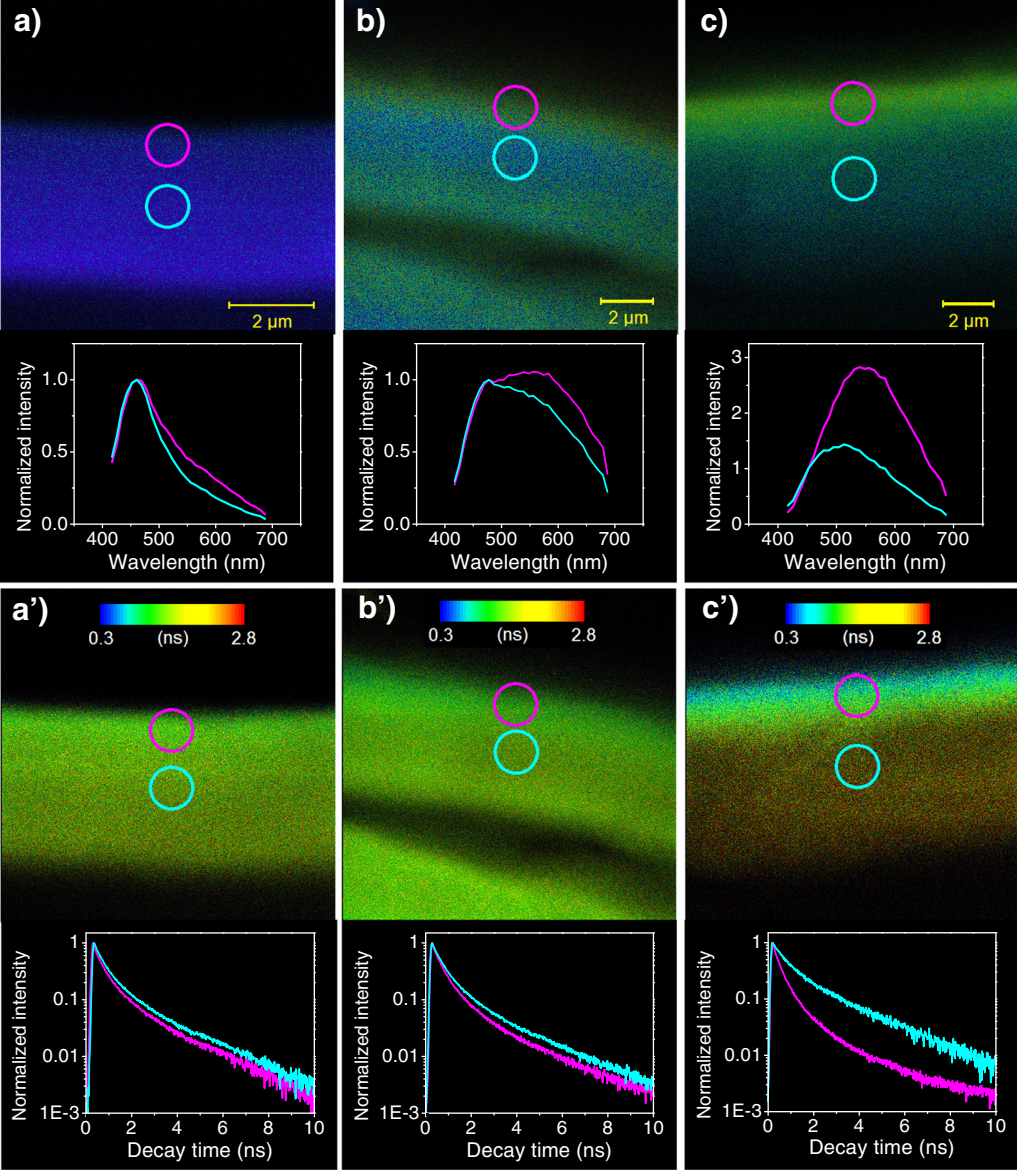
**Comparison among FLIM and spectral images of the cell wall for each pretreatment step.** Spectral images and fluorescence spectra for the regions indicated by the magenta spot (outermost) and the blue spot (innermost) of the cell wall of untreated bagasse (**a**), bagasse treated with H_2_SO_4_ 1% (**b**) and bagasse treated with NaOH 2% (**c**). FLIM images and evaluated decay dependences for the regions indicated by the magenta spot (outermost) and the blue spot (innermost) of the cell wall of untreated bagasse (**a’**), bagasse treated with H_2_SO_4_ 1% (**b’**) and bagasse treated with NaOH 2% (**c’**). The samples were excited by two photons at 770 nm. The 2P spectral and FLIM images were obtained at the same position and focal plane adjusted at the fiber maximum diameter. They represent a magnified region of the cell wall that evidences changes in the lignin distribution represented by color contrast due to spectral shifts and decay time modifications.

These results show therefore that lignin substructures definitely contribute to its emission and decay properties. In addition, the bagasse pretreatments also cause lignin redistribution in the biomass, resulting in significant spectral and decay changes that can be used to produce color contrast on the microscopy images of sugarcane cell wall. Therefore, we combined fluorescence scan microscopy and the associated fluorescence lifetime (FLIM) technique to obtain both spatial and chemical information from lignin in the cell wall as well as its redistribution and interaction at a submicrometer level upon pretreatments. Figure 6 compares highly magnified fluorescence and the corresponding FLIM confocal images with the associated spectral and decay data evaluated from specific regions of the cell walls of (a), (a’) untreated bagasse; (b), (b’) bagasse treated with 1% H_2_SO_4_ and (c), (c’) bagasse treated with 2% NaOH. These samples were excited by two photons at 770 nm. Each pixel of the confocal fluorescence image corresponds to a characteristic broad emission spectrum, but it is sensitive enough to provide information on the molecular concentration (accumulation) or on the chemical modification through the change of the 2P spectrum width. These important spectral changes finally affect the true color image resulting from the pixel-by-pixel overlapping of the spectrally resolved emission light. In the same way, this experiment demonstrates that the decay time depends consistently on the modifications of the lignin chemical environment and concentration, which is very suitable to produce a good contrast in the FLIM image.

The spectral fluorescence and FLIM images for the raw bagasse fiber presented in Figures 6a and 6a’, respectively, show well resolved internal and external boundaries of the cell wall and a homogeneous blue color distribution along the fiber confocal plane. To get more quantitative information about lignin homogeneity, the fluorescence decay and the spectral data were evaluated for two different regions of the spectral and FLIM image: one close to an external border (magenta spot) and another in the middle part of the cell wall (blue spot). The fluorescence spectra were normalized at 478 nm.

Raw bagasse results presented on Figure 6 show only a slight spectral broadening and a net decrease of 10% in the mean decay time when the region close to the outermost interface of the fiber is compared to its middle region. This is consistent with previous reports on the lignin enriched region in the cell wall boundaries of plants [7]. This scenario changes significantly for the bagasse treated with 1% H_2_SO_4_ (Figures 6b and 6b’). The large spectral broadening due to lignin self-interaction, as observed in molecular aggregates, transforms the color image to blue-greenish in the middle region and to green-yellowish close to both cell wall boundaries. When we compare with the native bagasse fiber, the acid treatment produces a reduction of 11% and 7% in the mean decay time evaluated near the outermost interface and in the middle of the fiber, respectively. According to Figure 4a, this corresponds to a variation of about 5% in the lignin fraction. In the case of bagasse treated with 2% NaOH, the mean decay time of the middle region becomes 22% longer (Figures 6c’) in comparison to the value measured for the acid treated sample, while it is 24% shorter at the outermost regions, contributing to the high color contrast of the FLIM image. This result suggests that a significant fraction of lignin has been removed from the inner part of the cell wall and that other lignin fraction segregates and accumulates on the wall external border. The redistribution of lignin has been observed with alkali treatment [9] and is supported by the microscope spectral image depicted in Figure 6c. In this figure, the inner part color of the cell switches to a green-bluish while the cell outermost region turns to yellowish. This color contrast is explained by spectral narrowing in the middle range as a consequence of the alkali lignin removal and by spectral broadening that still persists at the outer interface due to the polymer accumulation. In both cases, the spectral width is smaller than that observed after the acid treatment, as demonstrated in Figure 5.

Regarding the geometric characteristics of the fibers and the focal adjustments used in the CLSM and FLIM measurements, effects such as light reabsorption and scattering must be taken into account in the spectral and fluorescence decay analysis of the confocal images. The light that probes the inner part of the cell wall would find a longer path to get in (excitation) or to escape (emission) from the fiber than the excitation light or the emitted one near the outer edge of the cell wall.

In the case of the FLIM experiment (Figures 5 and 6) using 2P excitation, the fiber is transparent to the infrared light (770 nm) used, so light penetration and focusing are not to be concerned. For the emission excited along the confocal plane, our results also indicate that the effects related to the optical path inside the cell wall are not so important. Firstly, because the spectral and FLIM images of the cell wall for the non-treated bagasse (highly lignified sample in Figure 6a and 6a’) are surprisingly homogeneous along the focal plane considering the blue emission of lignin in these samples. Such blue emission is closer to the effective absorption edge of this molecule and, consequently, could suffer strong red shifts near the inner edge (longer propagation length) due to reabsorption and scattering effects. But this was not the case, as the image is spectrally homogeneous and do not present any color or intensity contrast. Secondly, Figure 5 also corroborated by the evidence that we do not observe significant changes of the high energy emission tail for all investigated samples, where 2P excitation was employed and the spectra were evaluated in the middle region of the cell wall. The spectral shifts observed in this figure are thus related to photophysical processes associated to lignin concentration and arrangements rather to those effects caused by optical propagation path. In the case of FLIM images in Figure 6, the emission was detected using channel 1, which actually probes wavelengths longer than 490 nm. Light emitted in this long wavelength range is also less susceptible to reabsorption and scattering effects. So, geometrical effects on FLIM contrast in terms of detected intensity and sensibility are not expected in Figure 6.

## Conclusions

Our results show that the time-resolved approach and the FLIM methodology are useful tools for probing lignin distribution in biomass. Despite the complex structure of sugarcane cell wall, these results also make evident a strong correlation between the decay time of the lignin fluorescence and its structural re-arrangement within the cell wall. Overall, greater fluorescence decay times are correlated with the lower concentration of lignin. The lignin fluorescence can be unambiguously separated into two components: one with the fast and another with the slow decay time.

Detailed analysis of the influence of lignin redistribution within the samples as a result of the alkali treatment on the two decay times indicated the presence of domains with distinct concentration levels of lignin. Of those, the ones with lower lignin content are effectively delignified by the alkali treatment, whereas the one with higher concentration is much more recalcitrant toward alkali and becomes susceptible to the treatment only at the highest concentrations of NaOH used in our study.

Our experiments indicate that in untreated bagasse, lignin is arranged in a loose manner with relatively weak interaction between lignin macromolecules. The acid treatment aggregates the non-solubilized lignin thus increasing a number of different nanoenvironments around each molecule whereas the alkali treatment solubilizes and removes lignin. An inhomogeneity of the cell wall, with high concentration of lignin along the external border is verified after the alkaline pretreatment.

## Methods

### Sugarcane bagasse treatment

Samples of sugarcane bagasse were treated as described in [1]. The treatment consisted of two subsequent steps: the first one using H_2_SO_4_ 1% (v/v), to remove hemicellulose mainly, and the second one with NaOH for delignification. This second step was performed at various NaOH concentrations from 0.25% up to 4% (w/v). After each step, the samples were filtered and the solid fraction was abundantly rinsed until reaching neutral pH, then oven dried at 60°C for 24 hours. Single fibers were selected after bagasse decantation in water.

### HPLC Methodology

The resulting average fractions of cellulose, hemicellulose and lignin of the fibers were determined by High Performance Liquid Chromatography (HPLC) for sugar cane bagasse pith, as previously described [1].

### Eucalyptus fibers treatment

The eucalyptus fibers were supplied by a paper company with 2% lignin content after standard delignification process with acid and xylanases treatment.

### Preparation of the lignin film

An aqueous suspension (0.5 g/l) of eucalyptus lignin was prepared and its pH was adjusted to 9 by adding NaOH aliquots. A drop of this suspension was placed on a hydrophobic cover slip and a homogeneous film was deposited due to the selective migration of the lignin molecules to the border of the water drop during its slow drying process [30].

### Confocal microscopy and FLIM

A Zeiss LSM 780 confocal microscope with a 405 nm diode laser and a Coherent Chameleon laser (Ti:sapphire) as excitation sources for one- (1P) and two-photons (2P), respectively, were used in the experiments.

The fibers dispersed in water were dried on cover slips and the images were obtained with a Plan-Apochromat objective lens (63X, numerical aperture 1.4, oil immersion). The lignin film was observed with a C-Apochromat objective lens (63X, numerical aperture 1.2, water immersion) in the opposite side of the cover slip. The images were obtained by the average of two scans. In all experiments, at least three isolated fibers for each treatment were studied and no appreciate variation was observed in the fluorescence properties among or within-sample single fibers.

As the fibers are almost cylindrical shaped, the focal plane was always adjusted in order to get the maximal fiber diameter along its confocal image, which means that the middle of the fiber was focused. This focal adjustment assures the same focal depth and the better optical contrast between lumen and cell wall. In addition, we tried to minimize topological artifacts (trying to preserve the maximal diameter condition) in the image by analyzing preferentially isolated fibers that lied down directly on microscope cover slip.

Considering the numerical aperture and the wavelength of excitation, the spatial resolution is approximately 200 nm. The optical zoom is 63x. A further digital zoom was used (1.7 in Figures 1a and 1b, 12.5 in Figures 6a and 6a’, 10.7 in Figures 6b and 6b’, 8.1 in Figures 6c and 6c’). The percentages of the lasers nominal powers were 1.2% (~20 μW) for the 405 nm laser (CW) for a 20x objective, 35% (~40 μW mean value) for a 405 nm pulsed laser, 20% (~200 mW mean value) for the 2P laser for a 63x objective.

For FLIM, the 405 nm laser was pulsed at 20 MHz and the 2P laser at 80 MHz. The fluorescence was divided by a beam splitter in two detecting channels of a PicoQuant system: channel 1 detecting the fluorescence above 490 nm; and channel 2, detecting below this value. The method used was the time correlated single photon counting (TCSPC) using avalanche detectors, which has a time response limited at about 100 ps. Two-exponential fit was used to adjust the fluorescence decay data. The choice of the fitting range was set by the software program (Time Trace Analysis by PicoQuant GmbH) by considering the decay part of the time dependent data according to optimal parameters.

The optical setup was adjusted to the best signal-to-noise ratio and fixed when different samples were compared in both CLSM and FLIM modes.

## Abbreviations

1P: One-photon; 2P: Two-photon; CLSM: Confocal laser scanning microscopy; CW: Continuous wave; FLIM: Fluorescence lifetime imaging microscopy; HPLC: High proficiency liquid chromatography; NMR: Nuclear magnetic resonance; TCSPC: Time correlated single photon counting.

## Competing interests

The authors declare that they have no competing interests.

## Authors’ contributions

VCC performed the confocal microscopy and FLIM experiments, their analysis and drafted the manuscript. CAR carried out the bagasse treatments and the determination of the chemical compositions and helped to draft the manuscript. FRC worked in the methodology and insights to the experiments. IP contributed to suggestions to the experiments, the analysis of the results and their discussion. FEGG coordinated the study, contributed to the microscopy and FLIM experiments, to the analysis of the results and in the improvement of the manuscript. All authors read and approved the final manuscript.
